# Giant presacral schwannoma presenting with constipation: a case report

**DOI:** 10.1186/1752-1947-6-285

**Published:** 2012-09-10

**Authors:** Lasitha Samarakoon, Amila Weerasekera, Rukman Sanjeewa, Sarath Kollure

**Affiliations:** 1 General Surgical Unit, National Hospital, Colombo, Sri Lanka

## Abstract

**Introduction:**

Schwannoma, otherwise known as a neurilemmoma, is a tumor arising from peripheral nerve sheaths. Although commonly noted in association with the eighth cranial nerve as intracranial acoustic neuroma, cases of schwannoma arising in other locations have been reported in the literature. These tumors usually cause symptoms as a result of their mass effect and, since they are benign, encapsulated and non-invasive tumors, complete surgical excision is considered curative.

**Case presentation:**

We report the case of a 46-year-old Sri Lankan man who presented to our facility with recent onset of difficulty evacuating his bowels. He was noted to have a giant presacral schwannoma on magnetic resonance imaging scan. The mass was surgically excised with improvement of our patient’s symptoms. A subsequent histopathological examination confirmed the presence of a benign schwannoma.

**Conclusions:**

Although schwannomas commonly occur in the extremities, a rare case of occurrence in the pelvis is reported here. Due to the limited space in the pelvis, the local mass effect may be the presenting feature of such a lesion and surgical excision is curative.

## Introduction

Schwannoma, otherwise known as a neurilemmoma, is a tumor arising from peripheral nerve sheaths. Although commonly noted in association with the eighth cranial nerve as intracranial acoustic neuroma, cases of schwannoma arising in other locations have been reported in the literature [1-7]. These tumors usually cause symptoms as a result of their mass effect and, since they are benign, encapsulated and non-invasive tumors, complete surgical excision is considered curative.

We report the case of a 46-year-old Sri Lankan man who presented to our facility with recent onset of difficulty evacuating his bowels. He was noted to have a giant presacral schwannoma on magnetic resonance imaging (MRI) scan. The mass was surgically excised. Following surgical excision of the schwannoma, our patient showed marked symptomatic improvement. A subsequent histopathological analysis revealed features compatible with a benign schwannoma, without nuclear polymorphism or necrosis.

## Case presentation

**Figure 1:**
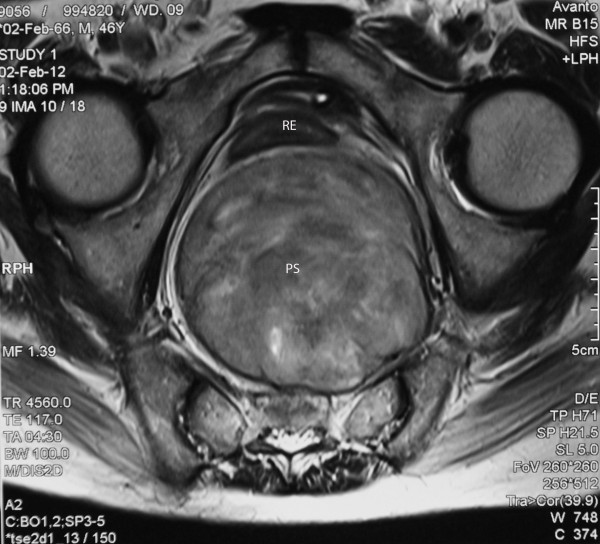
Magnetic resonance imaging of the presacral pelvic (PS) compressing the rectum (RE); transverse section.

**Figure 2:**
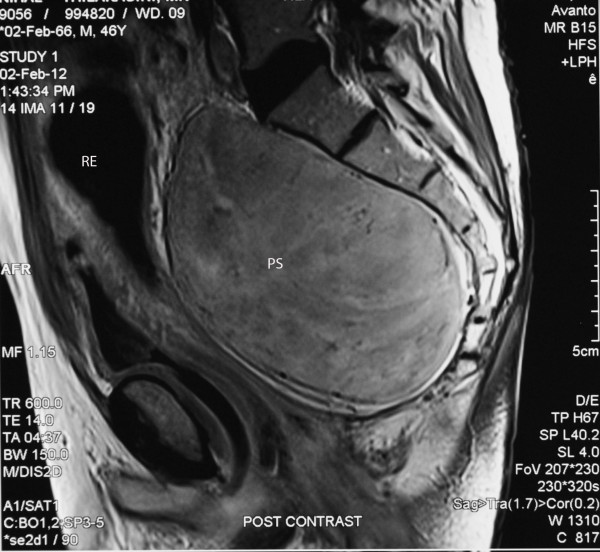
Magnetic resonance imaging of the presacral pelvic schwannoma (PS) compressing the rectum (RE); sagittal section.

A 46-year-old man presented to surgical unit of the National Hospital, Sri Lanka, with the primary complaint of difficulty in evacuating his bowels. He was paraplegic following laminectomy of the thoracic spine at the T4 to T8 level for removal of a lipoma in 1998, following which he had to rely on manual evacuation of the bowels. Recently, he was finding it exceedingly difficult to evacuate his bowels, and had sought medical advice. On examination, he was noted to have an empty rectum, and a mass was felt in the posterior rectal wall. Routine blood investigation results were within normal ranges. An ultrasound scan of the abdomen revealed a dilated sigmoid colon. Lower gastrointestinal endoscopy was attempted, which revealed external compression at 20cm of the sigmoid colon with a mass lesion posterior to the bladder. A contrast-enhanced computed tomography (CECT) scan of the pelvis showed a mass lesion in the presacral region, with a neurogenic origin. An MRI scan of the pelvic region revealed a presacral mass suggestive of a schwannoma causing anterior displacement of the rectum (Figures 1 and 2). A transrectal ultrasound scan (TRUS)-guided biopsy of the mass revealed that the histology was compatible with a schwannoma.

**Figure 3:**
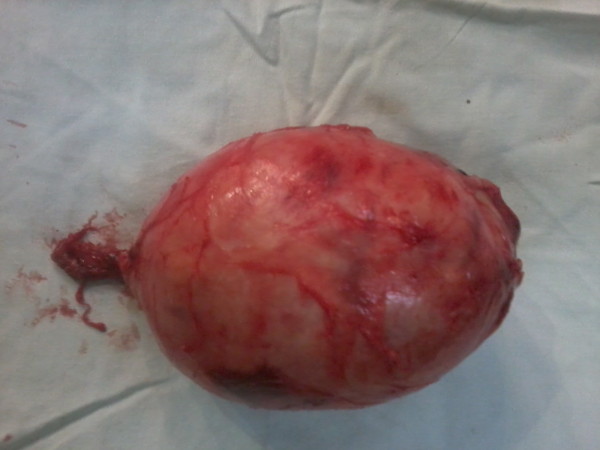
Macroscopic appearance of the resected specimen showing the smooth globular appearance of the schwannoma.

**Figure 4:**
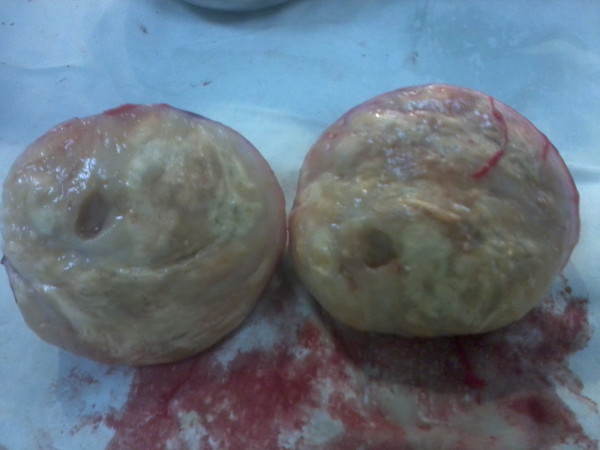
Cut section of the resected specimen.

Our patient underwent a laparotomy and excision of the presacral schwannoma. A globular mass measuring 1.4×9.0×8.0cm was removed (Figures 3 and 4). A meticulous dissecting technique was used to minimize bleeding during mobilization of the tumor and diathermy coagulation and local compression was used to arrest any bleeding that occurred. As a result, blood loss was minimal and postoperative blood transfusion was not required. The surgery was uneventful and there was marked symptomatic improvement in our patient after surgery. Subsequent histological analysis confirmed the diagnosis of a pelvic schwannoma. Suspicious histological features such as nuclear polymorphism or necrosis were not observed. Our patient was discharged from hospital and, since the histology was benign, surgical excision was considered to be curative.

## Discussion

Schwannomas are benign neoplasms arising from the nerve sheath. Although malignant schwannomas have been described, they commonly arise from the transformation of plexiform neurofibromatosis, and not from malignant degeneration of the schwannoma [8].

Schwannomas occur predominantly in men aged 20 to 50 years. Sites may vary, but they most commonly occur in the head and neck region [1-3] as well as flexor tendon sheaths of extremities [4]. Unusual locations such as the bladder [5], scrotum [6] and fallopian tubes have been reported [9]. Schwannomas occurring in the pelvis are rare and account for only 1% of cases [6]. Such tumors may present with non-specific pain, palpable mass or with rectal dysfunction [7].

Schwannomas may also occur within the lumen of the gastrointestinal tract. Peripheral nerve sheath tumors account for 2% to 6% of gastrointestinal stromal tumors (GISTs), with the most common location being the stomach and the small intestine [10]. The most common location for colorectal schwannomas is the caecum followed by sigmoid and rectosigmoid junction. Rectal bleeding, colonic obstruction, and abdominal pain were the most common presenting symptoms [11]. Such lesions occurring in the colon mimicking carcinoma [12,13], and coexistent schwannoma with synchronous colonic adenocarcinoma [14] have been reported. Very rarely, occurrence of schwannoma in the rectum has also been reported [15].

Since the tumor is characteristically non-infiltrative, well encapsulated and benign when extra-intestinal, the symptoms are usually due to the mass effect of the tumor. Most often detection is late, and tumors may grow to a very large size before being symptomatic. Large tumors (greater than 8 to 10cm) with cystic degeneration, calcification, interstitial fibrosis and calcification are termed to be the ‘ancient’ variant [6]. Antoni A (cellular and interlacing fascicles) and Antoni B (less cellular and myxoid), together with uniform staining for S100 protein characterize the histological appearance of a typical schwannoma [16]. Malignant schwannomas are large, infiltrating and characterized histologically by perineural and intraneural spread, lesional proliferation, herniation into the lumina of the vessels and nuclear palisading [17].

Although fairly distinct in clinical and histological presentation, pre-operative diagnosis of a schwannoma is not easy owing to a lack of distinguishing features on imaging studies such as ultrasound, CT or MRI between benign and malignant schwannomas as well as schwannomas and other soft tissue tumors such as fibrosarcomas and liposarcomas [18]. There are case reports where schwannomas have been misdiagnosed as psoas abscesses [19] and ovarian dermoid cysts [20]. In a large radiological series, it was noted that a smooth well-defined border, ovoid and spherical shape and location in the presacral region or the lower retroperitoneum were the most distinguishing features of primary abdominal or pelvic schwannomas [18]. Imaging studies may be useful in planning out therapeutic interventions. Pre-operative needle aspiration cytology is of doubtful value as specimens thus obtained are frequently insufficient, and cellular pleomorphism noted in degenerative areas may be misinterpreted as malignancy [21].

Although surgical excision is curative in cases of benign schwannoma, malignant schwannomas carry a poor prognosis as they are commonly resistant to chemotherapy and radiotherapy [22]. Even excision of benign intrapelvic and retroperitoneal schwannomas can be associated with massive bleeding if the tumor capsule is adherent to the presacral venous plexus [16], although blood loss was minimal in our patient. Recently, successful laparoscopic excision of a pelvic schwannoma [23-25] has also been reported.

## Conclusions

Although schwannomas commonly occur in the extremities, more rare sites of occurrence such as presacral locations should always be considered in the differential diagnosis of a pelvic mass. Such lesions may produce a significant mass effect due to the limited space available to accommodate growth in the rigid bony skeleton in the pelvis. Identification is important because surgical excision, either open or laparoscopic, is curative.

## Consent

Written informed consent was obtained from the patient for publication of this case report and any accompanying images. A copy of the written consent is available for review by the Editor-in-Chief of this journal.

## Competing interests

The authors declare that they have no competing interests.

## Authors’ contributions

LS gathered data and prepared the initial manuscript. AW and RS performed the surgery and made critical revisions to the manuscript. AW, RS and SK were in charge of the surgical care of our patient. SK supervised the project overall. All authors read and approved the manuscript.
